# Palmitic Acid Modulates Microglial Cell Response to Metabolic Endotoxemia in an In Vitro Study

**DOI:** 10.3390/nu15153463

**Published:** 2023-08-05

**Authors:** Mateusz Chmielarz, Beata Sobieszczańska, Andrzej Teisseyre, Magdalena Wawrzyńska, Edyta Bożemska, Kamila Środa-Pomianek

**Affiliations:** 1 Department of Microbiology, Wroclaw Medical University, 50-365 Wroclaw, Poland; mateusz.chmielarz@student.umw.edu.pl (M.C.); edyta.bozemska@umw.edu.pl (E.B.); 2 Department of Biophysics and Neuroscience, Wroclaw Medical University, 50-365 Wroclaw, Poland; andrzej.teisseyre@umw.edu.pl (A.T.); kamila.sroda-pomianek@umw.edu.pl (K.Ś.-P.); 3 Department of Preclinical Studies, Faculty of Health Sciences, Wroclaw Medical University, 50-365 Wroclaw, Poland; magdalena.wawrzynska@umw.edu.pl

**Keywords:** metabolic endotoxemia, microglial cells, palmitic acid, oxidative stress, inflammation

## Abstract

Metabolic endotoxemia (ME) is characterized by a 2–3-fold increase in blood endotoxin levels and low-grade systemic inflammation without apparent infection. ME is usually accompanied by metabolic syndrome, characterized by central obesity and hyperlipidemia. According to numerous studies, ME may lead to functional brain disorders, including cognitive decline, depression, and dementia. In the current in vitro study, we aimed to determine the direct and indirect impact of endotoxin (LPS) and palmitic acid (PA), representing saturated fatty acids, on the inflammatory and oxidative stress response in the human microglial HMC3 cells unstimulated and stimulated with IFNγ. The study’s results revealed that direct HMC3 cell exposition to endotoxin and PA increased inflammatory response measured as levels of IL-6 and MCP-1 released into the medium and PGE2 levels in cell lysates. Moreover, direct HMC3 cell treatment with PA and LPS induced oxidative stress, i.e., ROS and COX-2 production and lipid peroxidation. On the contrary, an indirect effect of LPS and PA on microglial cells, assessed as the impact of macrophage metabolites, was much lower regarding the inflammatory response, although still associated with oxidative stress. Interestingly, IFNγ had a protective effect on microglial cells, reducing the production of pro-inflammatory mediators and oxidative stress in HMC3 cells treated directly and indirectly with LPS and PA.

## 1. Introduction

The Western diet is characterized by an excessive supply of dietary calories in the absence of compensatory energy expenditure, leading to obesity. One of the factors influencing obesity is the gut microbiota, which in turn is modulated by the diet. A long-term high-fat diet (HFD) leads to gut dysbiosis, considered a quantitative and qualitative change in the microbiome. Gut dysbiosis induces subacute inflammation of the intestinal mucosa and increases intestinal barrier permeability, referred to as leaky gut, which exposes the host to toxic bacterial metabolites such as lipopolysaccharide (LPS, endotoxin) [1]. LPS at low concentrations can induce chronic inflammation, known as metabolic endotoxemia (ME) [2]. An HFD increases the abundance of Gram-negative bacteria in gut microbiota, the primary source of LPS, and enhances the level of LPS leaking through the intestinal barrier. It has been shown that plasma LPS levels are related to dietary fat content [3]. In HFD, LPS was elevated about 1.4 to 2.7 times the level determined in a balanced diet and is opposed to the LPS levels found during infection or septic shock, where endotoxin levels are 10–50 times higher [3,4]. Jeong et al. [5] showed that HFD increases plasma and fecal LPS levels and induces an increase in Gram-negative *Enterobacteriaceae*, well-known endotoxin producers. In the circulation, LPS mediates a TLR4-CD14-dependent immune response in the host organism. As a result of the LPS-activated TLR4-FAK-MyD88 pathway cascade, the activation of NF-kB occurs and increases gene expression of tumor necrosis factor-α (TNF-α), IL-6, inducible NO synthase (iNOS), and monocyte chemotactic protein-1 (MCP-1), which in turn can lead to brain microglia activation and cognitive impairment [6].

Moreover, dietary fat promotes endotoxin uptake from the gut. Studies by Ghoskal et al. [7] have shown that LPS is bound by enterocytes and transported to the Golgi apparatus, where chylomicrons are formed, transporting long-chain fatty acids through the mesenteric lymph and blood. The high affinity of LPS for chylomicrons facilitates its passage from the intestines into the lymph and blood. Hence, even in healthy and lean individuals, a fat-rich meal induces postprandial endotoxemia, as confirmed by numerous studies on volunteers [8,9,10]. Nevertheless, in lean, healthy individuals, postprandial endotoxemia lasts only a few hours and rarely, if ever, induces inflammation in contrast to obese individuals [11,12]. In obese patients, chronic endotoxemia is usually found, which may eventually contribute to functional changes in the brain [13,14,15].

The link between HFD and the risk of developing psychiatric and neuroinflammatory disorders is increasingly being pointed out. Adolescents suffering from depression have been shown to have a 70% higher risk of obesity, and in turn, obese adolescents present a 40% higher risk of depression [16]. High levels of endotoxin in the blood induce acute peripheral inflammation associated with releasing pro-inflammatory cytokines. Some of these cytokines can cross the BBB and induce the migration and accumulation of microglia cells around endothelial cells, thus enhancing the BBB [17,18]. On the contrary, chronic systemic inflammation, e.g., associated with ME decreases the BBB by transforming microglial cells to phagocytic phenotype, able to engulf the end-feet of astrocytes supporting BBB endothelial cells [18]. Numerous studies have indicated that microglia cells play a critical role in regulating the BBB directly and indirectly by low-grade inflammation in chronic endotoxemia [19]. Brain tissue is composed mainly of sphingolipids and cholesterol, which comprise about 25% of the brain’s dry weight. Hence, cholesterol and fatty acids (FAs) are essential in maintaining brain homeostasis as a vital component of cell membranes that maintain neuronal plasticity [20]. The brain cannot synthesize lipids, which must be supplied from the peripheral blood and penetrate the blood–brain barrier [21,22]. In CNS, FAs act as an essential mediator of metabolism and a rich energy source. However, the excessive supply of FAs, especially saturated fatty acids (SFAs), generates inflammation in the brain [23,24,25]. SFAs can affect the brain in various ways, such as increasing α-synuclein synthesis or stimulating the pro-inflammatory phenotype of microglia [22,26,27,28,29]. In vitro studies indicated that SFAs increase amyloid precursor proteins and β-amyloid (Aβ) aggregation [30,31,32].

Studies by Winocur et al. [33] on transgenic mice revealed that animals fed a Western diet for four months showed increased Aβ concentrations in the brain. According to these researchers, a long-term diet rich in saturated fatty acids disrupts glucose regulation, thus reducing glucose uptake in the brain’s hippocampal region. Karmi et al. [34] demonstrated that the uptake of fatty acids in the brains of obese patients with ME was elevated compared to healthy, lean subjects. Moreover, obesity impairs BBB integrity facilitating CNS infiltration with inflammatory cells and mediators and, thus, neuroinflammation [33,34,35,36]. Increased triglycerides and cholesterol levels in the blood of obese people are one of the parameters of ME that are concurrently a potential risk factor for cognitive impairment and dementia associated, among others, with AD [37].

Microglia are phagocytic cells in the brain that eliminate damaged neurons, apoptotic cells, or cellular substructures like synapses. Microglia respond to even slight changes in their local microenvironment, maintaining homeostasis in the brain [38]. Under physiological conditions, microglia play an essential role in neuronal survival by producing neurotrophic factors. Excessive activation of microglia by internal or external stimuli triggers the release of various pro-inflammatory neurotoxic factors, such as reactive oxygen species (ROS), tumor necrosis factor α (TNFα), interleukin-6 (IL-6), interleukin- 1β (IL-1β), and nitric oxide (NO), which, cause neuronal damage and enhance the progression of neurodegenerative diseases [39,40,41].

This in vitro study aimed to determine the direct and indirect effects of ME on the activation of human microglial cells of the HMC3 cell line. The direct impact of endotoxin and palmitic acid (PA) as the representative of SFAs, on HMC3 cells, corresponding to the passage of these substances via the blood–brain barrier (BBB), was investigated by the direct exposition of microglial cells to LPS and/or PA. The indirect effect, corresponding to the chronic systemic inflammation induced by ME, was studied by stimulating HMC3 cells with conditioned media derived from macrophages stimulated with LPS and PA at the same concentrations as those used in the direct effect study. In addition, to bring our research model closer to the in vivo situation, the direct and indirect effects of endotoxin and PA were studied on microglial cells pre-stimulated with IFNγ, produced peripherally by lymphocytes and locally during inflammation in the central nervous system by microglia and astrocytes. The HMC3 cell activation was determined based on pro-inflammatory cytokines, MCP-1, IL-6, and PGE2, and oxidative stress parameters, i.e., reactive oxygen species (ROS), cyclooxygenase-2 (COX-2), and malondialdehyde (MDA), indicative of lipid peroxidation

## 2. Materials and Methods

### 2.1. Chemicals Used in the Study

PA, fatty acids- and endotoxin-free bovine serum albumin (BSA), and LPS from *E. coli* O:55 were all obtained from Sigma-Aldrich (Germany, Darmstadt). Cell culture, fetal bovine serum (FBS), media, and supplements were all purchased at ThermoFisher Science (U.S., Waltham, Massachusetts). The human monocytic THP-1 cell line (EP-CL-0233) was purchased from Elabscience (U.S., Huston, Texas). ELISA kits for COX-2, MDA, and PGE2 detection were from Abcam, whereas ELISA kits for IL-6 and MCP-1 assessment were from Invitrogen (U.S., Waltham, Massachusetts). The 2′,7′-dichlorodihydrofluorescein diacetate (H_2_DCF-DA), MTT (3-[4,5-dimethylthiazol-2-yl]-2,5-diphenyltetrazolium bromide), FITC-phalloidin, and PMA (phorbol 12-myristate 13-acetate) were from Merc Life Science (Germany, Darmstadt).

### 2.2. Microglial Cell Culture

The study was performed on human microglial cells HMC3 cell line (CRL 3304) cultured in EMEM medium supplemented with 10% FBS and penicillin–streptomycin (10.000 U and 10 mg per mL, respectively) at 37 °C in 5% CO_2_ in a humid atmosphere. For the study, HMC3 cells were stimulated with 400 U/mL interferon γ (IFNγ) for 24 h to activate microglial cells.

### 2.3. Palmitic Acid Preparation

PA was dissolved to the concentration of 500 mM in NaCl and heated at 70 °C to obtain a clear solution. Fatty acids-free BSA was dissolved in an EMEM medium to receive a 10% solution and filter-sterilized. Then, PA was complexed with BSA, vortexed, and sonicated for 15 min at 55 °C twice. The final concentration of PA in BSA was 5 mM, and the PA to BSA ratio was 3.2:1. Next, the PA-BSA solution was split into small portions and frozen at −20 °C. A 10% BSA-NaCl solution was prepared as vehicle control. The PA solution was heated at 55 °C for 15 min before dilution to the appropriate concentration (200 µM) in the cell culture medium for the experiments.

### 2.4. Microglial Cell Viability and Morphology

The HMC3 cells were seeded at 5 × 10^4^ cells/mL density into a 96-well culture plate and incubated overnight to obtain an 80% monolayer. The cells were then stimulated with IFNγ at 400 U/mL for 24 h. After that, HMC3 cells were treated with LPS alone at 5 ng/mL, PA alone at 200 µM, or a combination of LPS and PA at the same concentrations for 20 h. The cell viability was measured with MTT. The MTT solution (0.5 mg/mL) in the cell culture medium was added to the cells and incubated for 3 h. Thereafter, the medium was discarded, and the insoluble purple formazan crystals were dissolved in DMSO with shaking for 5 min. The resulting-colored reaction was quantified by measuring absorbance at 570 nm in a multi-well spectrophotometer. Untreated MHC3 cells and microglial cells treated with PA solvent, i.e., BSA-NaCl, served as the negative and the vehicle control, respectively. The assay was performed twice in eight wells each. To determine the exact number of cells per 1 mL, a suspension of HMC3 cells at a density of 1 × 10^6^ cells per 1 mL was diluted in the wells of a microtiter plate and then incubated for 3 h with MTT as described above. A standard curve of cell viability to cell number was plotted from the results. The morphology of HMC3 cells growing in a 24-well culture plate with round glasses and stimulated with IFNγ, and then treated with PA and LPS, as described above, was determined in a fluorescence microscope after staining the cell’s actin-cytoskeleton with FITC-phalloidin.

### 2.5. Conditioned Media

THP-1 cell line was cultured in RPMI-1640 medium with 10% FBS and 1% antibiotics solution (penicillin, streptomycin). THP-1 cells were differentiated into monocyte-derived macrophages (MDM) with 50 ng/mL PMA for 48 h. After that, cells were matured for the next two days in a cell culture medium without PMA. The obtained MDMs were stimulated for 20 h with LPS and PA at 5 ng/mL and 200 µM concentrations, respectively. Next, the culture media from the above MDMs were drawn, centrifuged for 10 min at 1500× *g*, and filter-sterilized (0.22 µm). The resultant supernatants were frozen at −70 °C for further study. Since the conditioned media (CM) from macrophages apart from LPS and PA contain cellular metabolites and cytokines in concentrations toxic to cultured cells, CM were diluted before application to microglial cells in a cell culture medium to a concentration of 25%. Resultant CM were hereafter referred to as CM from MDM treated with LPS (CM-LPS), PA (CM-PA), or LPS and PA combined (CM-LPS + PA).

### 2.6. Oxidative Stress Assessment

The H_2_DCF-DA fluorescent probe was used to assess ROS production in HMC3 cells treated with PA and LPS for 20 h. HMC3 cells treated with 50 µM H_2_O_2_ served as a positive control. After treatment, cells were washed three times with Hank’s balanced solution (HBSS), and 10 µM H_2_DCF-DA in HBSS was added to cells for 30 min. The fluorescent product was quantified with a spectrophotometer, the Tecan Infinite M200 plate reader at 488/525 nm. Other oxidative stress parameters, i.e., COX-2 and its metabolite prostaglandin E2 (PGE2) and lipid peroxidation (MDA), were detected in HMC3 cell lysates. PGE2 and MDA were detected using immuno-enzymatic, colorimetric ELISA assays, whereas COX-2 was designated in a fluorometric assay. All assays were performed according to the producer’s instructions.

### 2.7. IL-6 and MCP-1 Assays

IL-6 and MCP-1 levels were assessed in a cell culture media collected from HMC3 cells treated with PA or/and LPS for 20 h, as well as in CM media derived from MDM treated for 24 h with LPS and/or PA at the same concentrations as those used for the direct HMC3 cell treatment using commercial human IL-6 and MCP-1 immuno-enzymatic kits ThermoFisher Science (U.S., Waltham, Massachusetts). The assays were performed according to the manufacturer’s instructions.

### 2.8. Statistical Analysis

All experiments were performed independently, three times in duplicate. *T*-test was used to determine the differences between the means of the two groups. The one-way analysis of variance (ANOVA) was used to determine the differences among groups, with *p*-value < 0.05 considered statistically significant. All data are presented as mean ± standard deviation.

## 3. Results

### 3.1. HMC3 Viability and Morphology

**Figure 1:**
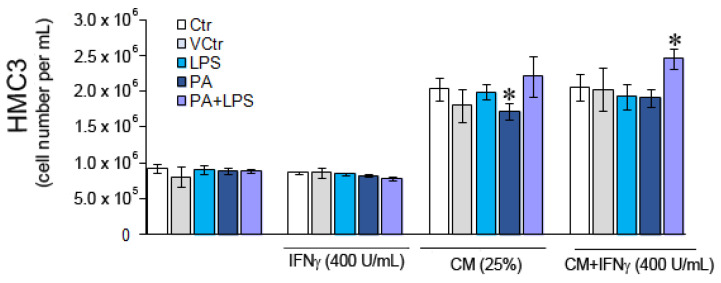
The viability and proliferation of HMC3 cells stimulated and unstimulated with IFNγ (400 U/mL) and treated directly with LPS, PA, and LPS + PA or indirectly with appropriate conditioned media (CM). Untreated cells and PA solvent (BSA-NaCl)—treated cells served as the negative control (NCrt) and the vehicle control (VCrt), respectively. * *p* < 0.05 indicates differences between unstimulated and IFNγ-stimulated cells treated with CM.

**Figure 2:**
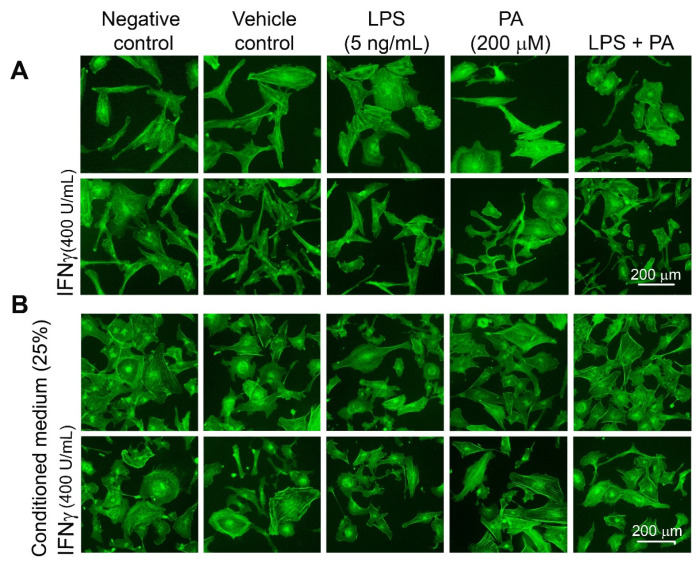
The morphology of HMC3 cells stimulated and unstimulated with IFNγ (400 U/mL) and treated directly with LPS, PA, and LPS + PA (**A**) or indirectly with appropriate CM (**B**). Fluorescence microscope images after staining cell’s actin-cytoskeleton with FITC-phalloidin.

To establish the impact of direct and indirect treatment of LPS and PA on unstimulated and IFNγ-stimulated HMC3 cells, MTT test results in the form of absorbance intensity were converted to the number of cells based on the standard curve. The direct treatment of HMC3 cells stimulated or unstimulated with IFNγ and then treated with PA or/and LPS for 20 h had no significant impact on cell viability and proliferation (Figure 1). On the other hand, CM enhanced the growth of microglial cells about two times (*p* < 0.05). These results indicated that the MDM metabolites present in CM augmented microglial cell proliferation. IFNγ had little, although statistically significant, stimulatory effect on cells treated with CM-PA (*p* = 0.009) and with CM-LPS + PA (*p* = 0.03) but not CM-LPS (*p* > 0.05). The untreated and unstimulated HMC3 cells presented spindle cell morphology, which was more prominent upon stimulation with IFNγ (Figure 2A). The cells became rounded upon being treated with LPS and PA, indicating their activation (Figure 2A). Similar rounded HMC3 cells were observed after the treatment of HMC3 cells with appropriate CM (Figure 2B).

### 3.2. Palmitic Acid and Endotoxin Modulate Cytokine Secretion by Microglial Cells

The inflammatory activation of microglial cells treated directly with LPS and/or PA and indirectly with corresponding CM, i.e., CM-LPS, CM-PA, and CM-LPS + PA, was studied based on the secretion of pro-inflammatory cytokine IL-6 and chemokine MCP-1, and PGE2. The untreated and vehicle control-treated HMC3 cells produced measurable levels of IL-6 (Figure S1A,B). Taking into consideration measurable levels of the cytokine in controls, as well as the strong correlation between the HMC3 cell number and the IL-6 and MCP-1 levels (Pearson’s correlation coefficient of 0.91 and 0.82, respectively; *p* < 0.00001), first, the cytokines levels measured in the appropriate CM were subtracted from determined cytokine levels in cell culture media from HMC3 cells treated indirectly with CM, and then normalized to cell numbers. Eventually, the ratios of cells treated directly and indirectly with PA and LPS to appropriate controls were calculated. The levels of IL- 6 and MCP-1 detected in the cell culture media from directly and indirectly treated HMC3 cells and expressed as pg per mL are presented in the Supplementary Materials (Figure S1). The results were further analyzed based on ratios to controls to make uniform the results for all inflammatory markers.

**Figure 3:**
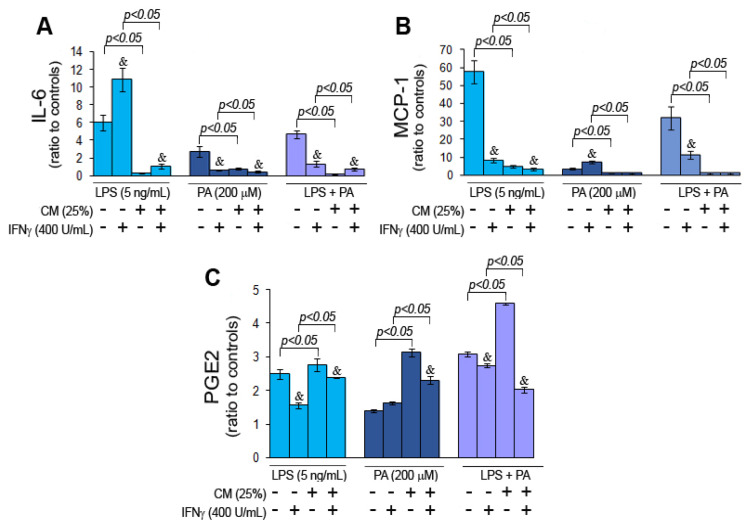
Inflammatory markers, i.e., IL-6 (**A**), MCP-1 (**B**), and PGE2 (**C**) in HMC3 cells stimulated and unstimulated with IFNγ and then treated directly with LPS and/or PA or indirectly with CM-LPS, CM-PA, and CM-LPS + PA. Statistically significant differences vs. controls ^&^ *p* < 0.05; unstimulated vs. IFNγ-stimulated cells and *p* < 0.05.

HMC3 cells directly exposed to LPS, PA, and LPS + PA produced significantly higher IL-6 levels than the corresponding controls (Figure 3A). In contrast, indirect HMC3 cell treatment with CM had no remarkable impact on IL-6 secretion compared to the controls. IFNγ significantly increased IL-6 levels only in cells treated directly with LPS compared to unstimulated cells. Similarly, direct HMC3 cell exposure to LPS and LPS + PA significantly impacted MCP-1 secretion, while direct cell treatment with PA had little effect on MCP-1 production (Figure 3B; Figure S1C,D). Indirect cell treatment with corresponding CM had no essential impact on MCP-1 compared to controls, just like cell stimulation with IFNγ. In contrast to IL-6 and MCP-1, direct HMC3 cell treatment with LPS, PA, and LPS + PA considerably increased PGE2 levels, while indirect treatment of HMC3 cells with appropriate CM even more increased PGE2 levels compared to directly treated cells (Figure 3C; Figure S1E). IFNγ, as in previous inflammation markers, decreased PGE2 production in directly and indirectly treated cells. Altogether, these results revealed that indirect cell treatment with CM had considerably less effect on IL-6 and MCP- production than direct HMC3 exposition to LPS and/or PA. However, indirect HMC3 cell treatment increased intracellular PGE2 synthesis. IFNγ had variable effects on inflammatory markers studied but generally decreased the inflammatory response of microglial cells, except cells directly exposed to LPS.

### 3.3. Palmitic Acid and Endotoxin Induce Oxidative Stress in Microglial Cells

**Figure 4:**
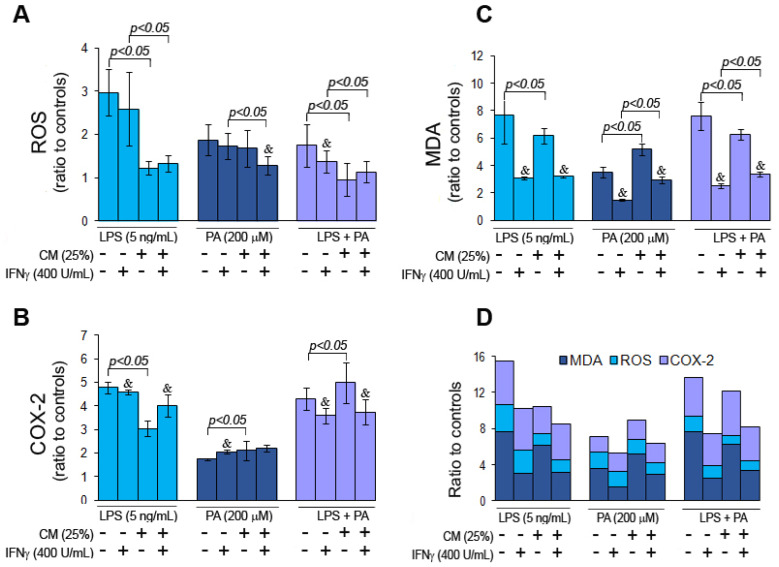
Oxidative stress markers, i.e., ROS (**A**), COX-2 (**B**), and MDA (**C**) in HMC3 cells stimulated and unstimulated with IFNγ and then treated directly with LPS and/or PA or indirectly with CM-LPS, CM-PA, and CM-LPS + PA. Comparison of ratio to controls of all oxidative stress markers (**D**). Statistically significant differences vs. controls ^&^ *p* < 0.05; unstimulated vs. IFNγ-stimulated cells and *p* < 0.05.

The direct and indirect roles of ME on oxidative stress in microglial cells were assessed by determining the levels of ROS, COX-2, and lipid peroxidation based on malondialdehyde (MDA) detection. Taking into account the effect of CM and IFNγ on the oxidative stress markers in both controls, i.e., negative and vehicle control (Figure S2), as well as the positive correlation of ROS levels with cell number (Pearson’s correlation coefficient 0.6; *p* = 0.005), the arbitrary fluorescence units (RFU) were normalized to cell number, and then the results were related to appropriate controls. Based on the positive correlations between ROS and COX-2 (Pearson’s correlation coefficient 0.72; *p* = 0.0003) and between COX-2 and MDA (Pearson’s correlation coefficient 0.91; *p* < 0.00001) to make uniform, the results for all results of oxidative stress markers were analyzed as ratios to the corresponding controls. The ROS levels in RFU, COX-2 at pg per mg proteins, and MDA at nmol per mg proteins are presented in the Supplementary Materials (Figure S2). The analysis demonstrated that direct HMC3 cell treatment with LPS induced higher ROS levels than PA and LPS + PA (Figure 4A). Corresponding CM decreased ROS levels by 1.4- fold (*p* = 0.0008) for CM-LPS and by 0.8-fold (*p* = 0.004) for CM-LPS + PA compared to directly treated cells, but the decrease was negligible for CM-PA (*p* > 0.05). IFNγ insignificantly decreased ROS levels in cells treated directly with LPS and PA, but the drop was significant in cells directly treated with LPS + PA. Moreover, in HMC3 cells treated indirectly with CM-LPS and CM-LPS + PA, IFNγ had no impact on ROS production but significantly decreased ROS in cells treated with CM-PA.

LPS and LPS + PA induced higher COX-2 levels than PA in directly treated cells (*p* = 0.00001 and *p* = 0.009, respectively) (Figure 4B). Indirect HMC3 cell treatment with the corresponding CM, however, had a variable impact on COX-2 production as it decreased its level (*p* = 0.02) in cells treated with CM-LPS, increased COX-2 in cells treated with CM-LPS + PA (*p* = 0.004), and did not affect cells treated with CM-PA (*p* > 0.05). HMC3 cell stimulation with IFNγ lowered COX-2 production in cells treated directly and indirectly with LPS and LPS + PA. In contrast, IFNγ increased COX-2 levels in cells treated with PA but did not affect cells treated indirectly with CM-PA.

Moreover, the levels of PGE2, a metabolite of COX-2, correlated with COX-2 levels in HMC3 cells (Pearson’s correlation coefficient 0.84; *p* < 0.00001).

The direct and indirect treatment of HMC3 cells with LPS, PA, and LPS + PA and corresponding CM increased MDA levels in unstimulated cells (Figure 4C). The stimulation of HMC3 cells with IFNγ decreased MDA levels significantly in directly and indirectly treated microglial cells.

A compilation of the assayed oxidative stress parameters showed that LPS alone or in combination with PA, and to a lesser extent, CM-LPS and CM-LPS + PA had the most significant effect on inducing oxidative stress in microglia cells compared to cells treated with PA alone (Figure 4D). Generally, IFNγ reduced oxidative stress in cells treated directly and indirectly with LPS and/or PA.

## 4. Discussion

Metabolic endotoxemia (ME) is characterized by a 2–3-fold increase in blood endotoxin levels and low-grade systemic inflammation without apparent infection [3,15,42]. ME is usually accompanied by metabolic syndrome, which, in addition to endotoxemia, is characterized by central obesity and hyperlipidemia [33]. ME is a well-known cause of cardiovascular disease but is also linked to neurological disorders [34].

Numerous laboratory and animal studies indicated that ME is associated with functional brain disorders, including cognitive decline, depression, and dementia [14,15,35,43,44,45]. However, there are few in vitro studies on the effects of ME associated with low, clinically irrelevant levels of endotoxin in the blood with associated hyperlipidemia on human microglial cells. Previously, Lu et al. [29], in an in vitro study on HMC3 cells, demonstrated that low doses of LPS combined with PA upregulated proinflammatory cytokines through MAPK, NFκB, and AP-1 signaling pathways.

This study examined two scenarios to better fit our in vitro model to ME in vivo. The first scenario assumed that ME-induced systemic chronic inflammation contributes to lowering the BBB, allowing small doses of LPS and PA to penetrate the CNS and directly affect microglia. The second scenario, assuming the indirect effect of ME on microglia, assessed the impact of ME-induced peripheral inflammation on microglia, taking the metabolites released by ME-responsive immune cells, i.e., monocytes/macrophages.

According to the first scenario, our findings showed that LPS, alone or combined with PA, had the most potent effect on the inflammatory and oxidative stress parameters. Interestingly, IFNγ decreased inflammatory and oxidative stress responses in HMC3 cells directly exposed to LPS and/or PA. These results partially corroborate the results of Lu et al. [29], who demonstrated that PA + LPS induced higher IL-6 levels than every compound individually. However, in contrast with Lu et al. [29], our study demonstrated that direct HMC3 cell exposure to LPS evoked more potent IL-6 and MCP-1 release compared to PA, which most probably resulted from a different way of analyzing the results which in our study took into account cell counts and controls differently for LPS and PA. Moreover, our studies and those of Lu et al. [29] differed with PA solvents (NaCl vs. alcohol). The co-toxicity of LPS and PA demonstrated in our studies and those of Lu et al. [29] was also confirmed by Wang et al. [46], who demonstrated that PA amplified LPS-induced MCP-1 production in macrophages.

Following the second scenario, our findings demonstrated that indirect HMC3 cell treatment with conditioned media had a lower effect on microglial cells than direct treatment. According to the study of Tucureanu et al. [47], conditioned media derived from MDM stimulated with endotoxin contained a mixture of pro-inflammatory and anti-inflammatory cytokines and chemokines, including IL-1, IL-6, IL-8, IL-10, IL-12, IL-15, TNFα, MCP-1, TGFβ, which affect microglia activation. In the study, we determined only the levels of IL-6 and MCP-1 in CM from MDM stimulated with LPS and/or PA, and both these inflammatory mediators were secreted by MDM in high concentrations. However, the conditioned media rich in these and other cytokines, chemokines, and growth factors had little or no effect on inflammatory response in microglial cells. In contrast, conditioned media affected oxidative stress parameters, i.e., MDA and COX-2 levels in HMC3 cells.

Lipid peroxidation is a self-propagating chain reaction that can lead to severe tissue damage [48]. Hence, even a few oxidized lipid molecules in the microglia milieu can initiate a cascade of membrane lipid peroxidation and irreparable damage to microglial cells, resulting in inflammation. Barger et al. [49] demonstrated that lipid peroxidation in microglia contributes to glutamate release, one of the excitotoxins evidencing neurotoxicity. Similar to direct treatment, IFNγ decreased the indirect effect of CM-LPS and/or CM-PA on microglial cells. This suggests that ME-induced peripheral inflammation would have little effect on microglia, even if the metabolites secreted by macrophages were to penetrate the BBB. This corroborates the findings of Andree et al. [8] regarding postprandial endotoxemia, which, although associated with an increase in endotoxin (and fatty acid) levels in the blood of subjects examined and induced the release of proinflammatory cytokines, aside from causing transient sickness-like behavior in some individuals, fundamentally it did not induce neuroinflammation. These results also align with studies of Lively et al. [30] on primary rat microglia exposed to LPS or IFNγ with TNFα, where LPS evoked higher proinflammatory gene expression than proinflammatory stimulus, i.e., IFNγ with TNFα.

Endotoxin is a well-known factor inducing inflammation and oxidative stress via the activation of NFĸB in many cell types [50,51]. Similarly, SFAs, including PA, are considered inflammatory mediators. Raman et al. [52] demonstrated that palmitate alters the expression and COX-2 function in vascular smooth muscle cells. According to their study, palmitate increased total COX-2 protein levels and enhanced the iso-enzyme glycosylation to the more active form by regulating its post-translational modification. According to Zhou et al. [53], PA excess induced neuroinflammation signaling pathways associated with microglia activation and neurodegeneration.

The inflammatory mediators elicited by endotoxin and PA assessed in the study included proinflammatory cytokine IL-6, chemokine MCP-1 (CCL-2), and prostaglandin PGE2, which are all important in the CNS and neurodegenerative diseases. IL-6, which is present in low levels in healthy human brains, plays a pleiotropic role in CNS homeostasis and normal functions, i.e., neurogenesis, neuron excitability and phenotype, sleep regulation, learning, and memory [54,55,56]. The dysregulated IL-6 production is associated with cognitive and memory dysfunction, neuroinflammation, and deposition of the precursor of β-amyloid protein and is associated with schizophrenia, major depression, bipolar disorders, AD, and others [56]. MCP-1 is expressed in the CNS and plays an essential role in the activation and proliferation of microglial cells and their migration to endangered or damaged sites. In addition, MCP-1 promotes the loss of BBB integrity by destroying adherent junctions and offering a route for infiltrating inflammatory cells and mediators. A correlation has been shown between MCP-1 plasma levels and the rate of cognitive decline in AD and Parkinson’s disease progression. Moreover, MCP-1 levels in CSF correlate with tau protein and Aβ levels [6,57,58,59]. It has also been demonstrated that MCP-1 plays a role in senile memory decline. In asymptomatic elderly individuals, the decline in verbal episodic memory worsened over time and correlated with an increase in MCP-1 levels. Finally, prostaglandin PGE2 is a crucial mediator of inflammation, regulating apoptosis, angiogenesis, and cell proliferation. Under physiological conditions, PGE2 is released in the brain by tanacytes, astrocytes, and neurons, and under inflammatory stimuli, also by microglial cells and vascular endothelial cells [60]. By activating E-prostanoid receptors EP1 through EP4, PGE2 activates inflammatory signaling pathways contributing to neurotoxicity [61]. Taking into consideration the involvement of IL-6, MCP-1, and PGE2 in neurotoxicity, the finding of our study suggested that the direct impact of ME on microglia may pose a risk for the development of neurodegeneration, especially during long-term stimulation of microglia. However, despite PGE2 involvement in the classical pro-inflammatory response, it can also act as an immunosuppressor, down-regulating microglial activation [62]. Boje et al. [63] reported that inhibition of prostaglandin synthesis in experimental meningitis exacerbated meningeal NO production and toxicity and failed to prevent BBB destruction. Studies by Ajmone-Cat et al. [62] and Kuo et al. [64] showed that long-term stimulation with LPS led to microglia tolerance to endotoxin. According to the study of Ajmone-Cat et al. [61], elevated synthesis of PGE2 after multiple LPS challenges led to decreased synthesis of NO and TNFα by down-regulating NFĸB signaling. In line with these authors, COX-2/PGE2 pathway activation prevents microglia overstimulation from continuous exposure to the same danger signals and indicates the immunosuppressive role of PGE2 in microglia. On the other hand, considering the modulatory effect of PA on microglia cells’ response to LPS demonstrated in the study, as well as that PA itself activates NFĸB, it is unknown whether LPS combined with PA would induce microglia tolerance as well. Nevertheless, microglia tolerance to endotoxin raises questions about the deleterious effects of persistent ME in obese individuals. Despite this, the simultaneous long-term effect of LPS and SFAs on microglia requires further clarification, even more so since our results showed that PA modulated the microglia response to endotoxin, e.g., PA combined with LPS decreased ROS, IL-6, and MCP-1 levels in HMC3 cells but had no effect on COX-2 and MDA levels. These results indicated that PA itself may inhibit some pathways in microglial cells while increasing others.

Moreover, our findings demonstrated that IFNγ attenuated oxidative stress and inflammatory response to endotoxin and PA in microglial cells.

IFNγ is a pleiotropic cytokine involved in modulating innate and acquired immune mechanisms. In addition to its well-recognized pro-inflammatory function, IFNγ also plays a vital role as an anti-inflammatory and protective factor, particularly in the CNS [65,66]. The binding of IFNγ to the IFNγR receptor, expressed on most cells, including microglia, activates receptor-related JAK1 and JAK2 Janus protein kinases, followed by tyrosine phosphorylation and STAT1 activation, which travels to the nucleus and directly activates transcription of IFNγ-stimulated genes (ISGs). These genes affect effector immune functions, including MHC antigen-presenting molecules, cytokines, chemokines, and phagocytosis receptors [66]. Applying gene array analysis and selecting specific genes involved in the immune response, Shaked et al. [67] compared the phenotypes of a rat’s microglia stimulated with LPS and IFNγ. This analysis showed that IFNγ signaling in microglia focused on the JAK/STAT pathway up-regulating STAT-1, while LPS signaling focused on NFĸB. Furthermore, LPS signaling was self-propagating via up-regulation of its receptor CD14. Moreover, in contrast to IFNγ, LPS increased in microglia mRNA expression of numerous pro-inflammatory cytokines, e.g., TNFα, IL-1a, IL-1b, IL-6, chemokines, like GRO, MCP-1, MIP-1a, MIP-1b, MIP-2, and enzymes COX-2, iNOs, MMP-9, and MMP-13. Likewise, Rock et al. [68] demonstrated that specific activation of JAK/STAT signaling in human microglia is moderated through a feedback mechanism that includes, among others, the suppression of IFNγ receptor elements and increased expression of STAT-induced inhibitor-1 (SSI-1), which in turn, by inhibiting STAT phosphorylation suppresses cytokine production. Additionally, IFNγ also regulates the production of anti-inflammatory cytokines, e.g., IL-4, IL-10, and IL-13 promoting the resolution of inflammation and through JAK/STAT pathway activation reparative phenotype of macrophages [69]. Studying experimental autoimmune encephalitis (EAE) in an animal model, Sosa et al. [70] showed that IFNγ stimulated microglia to break down oxidized lipids, preventing oxidative brain damage. The protective, antioxidant effect of IFNγ may be due to the ability of this cytokine to activate the kynurenine pathway. Christensen et al. [71] showed that one of the metabolites of the kynurenine pathway, i.e., 3-hydroxy anthranilic acid, is a potent antioxidant and free radical scavenger, which would explain the protective antioxidant role of IFNγ.

Our study has several limitations. First, our research involves only one selected concentration of LPS, PA, and IFNγ. Furthermore, we did not use time-course treatment, which would be valuable to determine whether LPS combined with PA could still induce microglia tolerance to LPS. Nevertheless, our study provided significant observations concerning the direct and indirect effects of ME on human microglial cells stimulated and unstimulated with IFNγ.

The increase in the incidence of neurodegenerative diseases, depression, and other functional brain disorders, as well as obesity associated with HFD rich in processed foods, strongly suggests an impact of diet on the gut microbiota and brain homeostasis, i.e., the brain–gut axis. Chronic ME accompanying metabolic syndrome in obese individuals and resulting from gut dysbiosis is not only a source of endotoxemia. During dysbiosis, many other bacterial metabolites can leak from the gut into circulation and affect the brain directly or indirectly. Moreover, even in clinically insignificant concentrations, bacterial metabolites can interact with each other or dietary components such as fatty acids and show increased toxicity. A detailed understanding of these relationships can help understand the pathomechanisms of neurological conditions and undertake targeted therapy.

## 5. Conclusions

The study’s results demonstrated that the direct impact of LPS and PA on HMC3 cells increased inflammatory response measured as levels of IL-6 and MCP-1, and PGE2. Moreover, direct treatment of HMC3 cells with PA and LPS induced oxidative stress, i.e., ROS and COX-2 production and lipid peroxidation. On the contrary, an indirect effect of LPS and PA assessed as the impact of macrophage metabolites on microglial cells was much lower regarding the inflammatory response, although still associated with oxidative stress in HMC3 cells. In addition, our results showed that IFNγ had a protective effect on microglial cells, reducing the production of pro-inflammatory mediators and oxidative stress in HMC3 cells treated directly and indirectly with LPS and PA.

## Data Availability

All data is available upon request from the corresponding author.

## Supplementary Materials

The following supporting information can be downloaded at: https://www.mdpi.com/article/10.3390/nu15153463/s1, Figure S1: Inflammatory markers (IL-6, MCP-1, and PGE2) in HMC3 cells. Figure S2: Oxidative stress markers (ROS, COX-2, and MDA) in HMC3 cells.
