# Supplementary material for: Palmitic Acid Modulates Microglial Cell Response to Metabolic Endotoxemia in an In Vitro Study
Source: Nutrients. 2023 Aug 5;15(15):3463. doi: 10.3390/nu15153463 (PMC10421407; doi:10.3390/nu15153463)
Supplement: Supplementary file 1 [file nutrients-15-03463-s001.zip › nutrients-2495391-supplementary.pdf]

Supplementary Materials

Inflammatory response of HMC3 cells unstimulated and IFNγ-stimulated to LPS and/or PA was investigated with immunoenzymatic assays based on IL-6 and MCP-1 secretion to the cell culture media, whereas PGE2 was detected in cell lysates. The levels of IL-6, MCP-1, and PGE2 detected are presented in Figure S1.

Oxidative stress in HMC3 cells unstimulated and IFNγ-stimulated was assessed based on ROS levels (assayed as fluorescence intensity after staining cells with an H2DCF-DA fluorescent probe), COX-2, and MDA (assayed using immunoenzymatic, colorimetric ELISA tests). The ROS, COX-2, and MDA levels are presented in Figure S2.

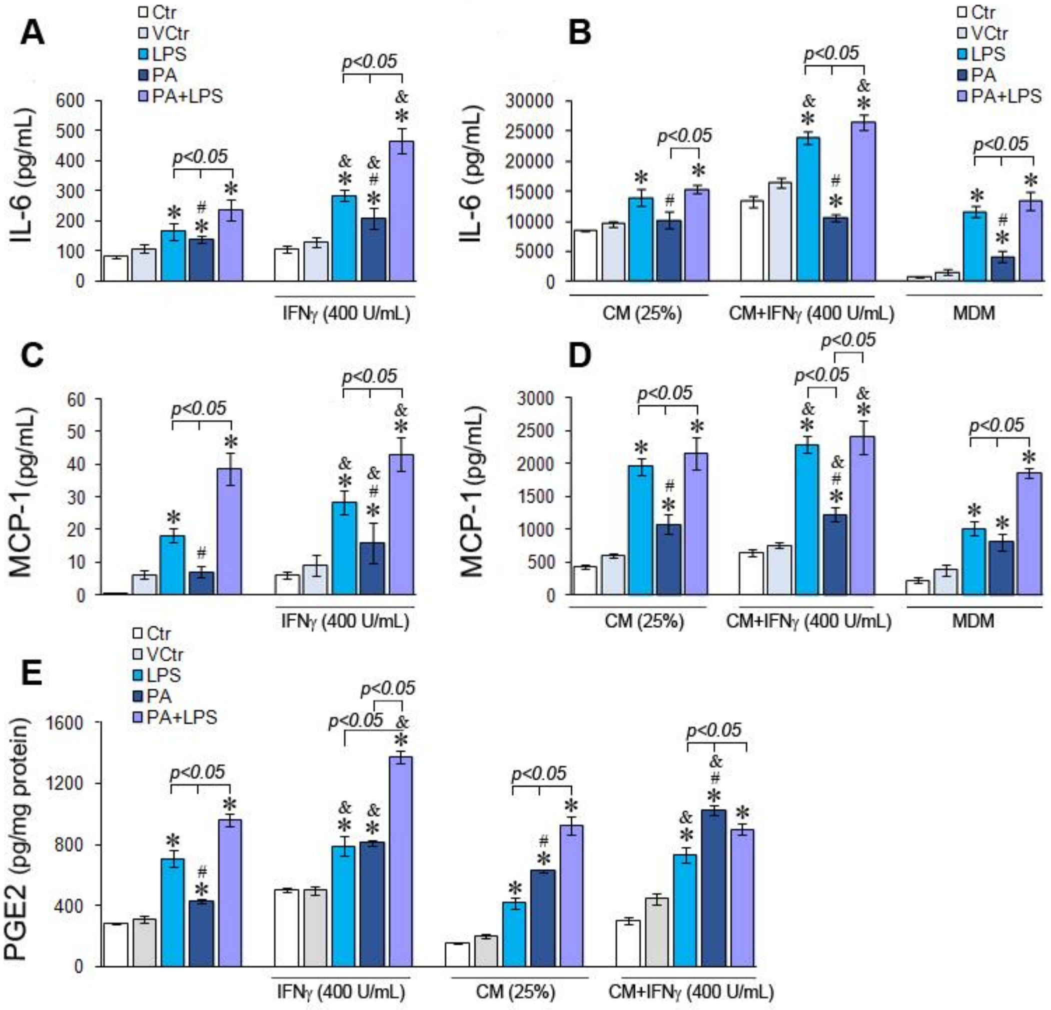

Figure S1. Inflammatory markers (IL-6, MCP-1, and PGE2) in HMC3 cells stimulated and unstimulated with IFNγ and then treated directly with LPS and/or PA or indirectly with CM-LPS, CM-PA, and CM-LPS+PA. Untreated cells were a negative control (NCrt) for HMC3 cells treated with LPS alone. The cells treated with PA solvent (BSA-NaCl) were the vehicle control (VCrt) for HMC3 cells treated with PA alone or combined with LPS. Statistically significant differences vs. controls * $p<0.05$; vs. LPS # $p<0.05$, unstimulated vs. IFNγ-stimulated cells & $p<0.05$.

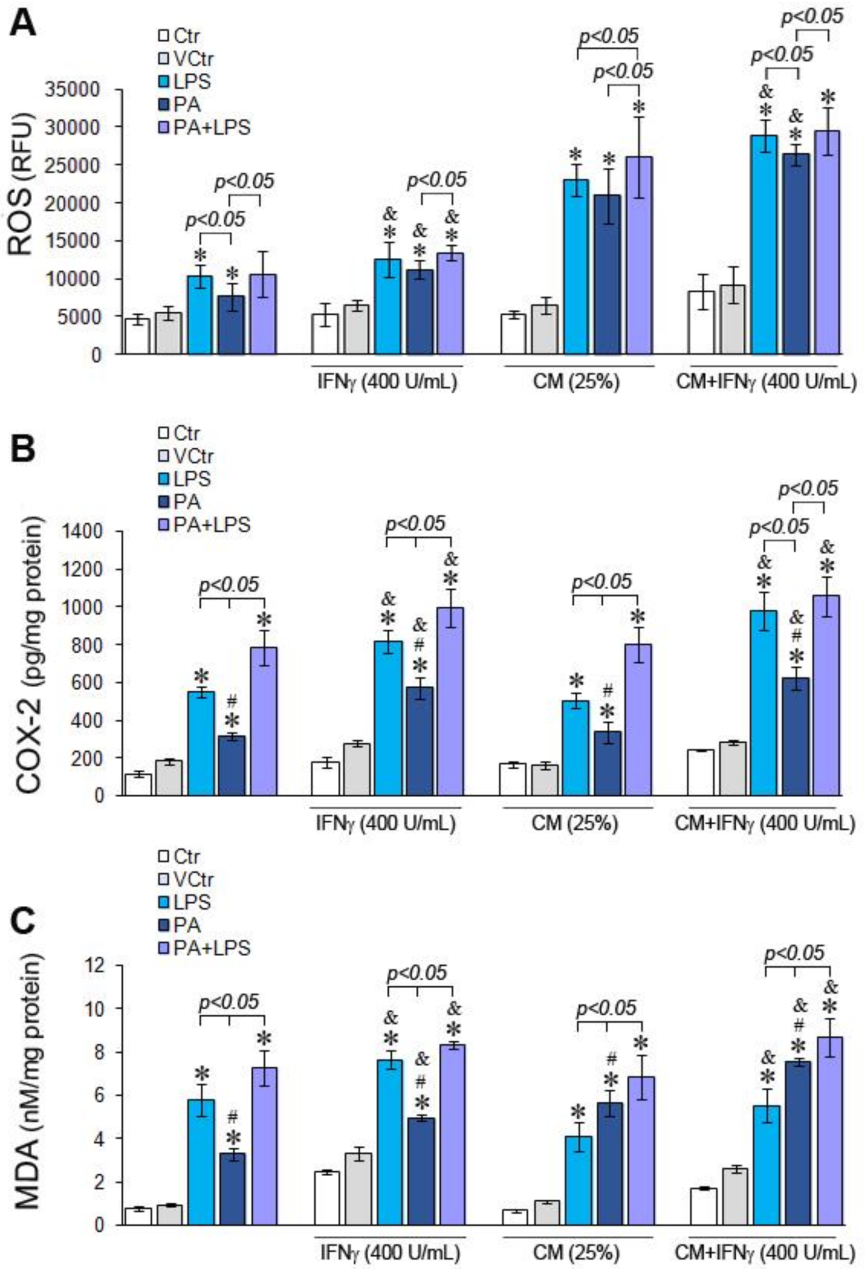

Figure S2. **Oxidative stress markers (ROS, COX-2, and MDA) in HMC3 cells** stimulated and unstimulated with IFNγ and then treated directly with LPS and/or PA or indirectly with CM-LPS, CM-PA, and CM-LPS+PA. Untreated cells were a negative control (NCrt) for HMC3 cells treated with LPS alone. The cells treated with PA solvent (BSA-NaCl) were the vehicle control (VCrt) for HMC3 cells treated with PA alone or combined with LPS. Statistically significant differences vs. controls * $p<0.05$; unstimulated vs. IFNγ-stimulated cells & $p<0.05$.
